# Intrahepatic cholestasis of pregnancy – Time to redefine the reference range of total serum bile acids: A cross‐sectional study

**DOI:** 10.1111/1471-0528.17174

**Published:** 2022-04-22

**Authors:** Mor Huri, Viola Seravalli, Camilla Lippi, Lorenzo Tofani, Andrea Galli, Felice Petraglia, Mariarosaria Di Tommaso

**Affiliations:** ^1^ Obstetrics and Gynaecology Unit, Department of Health Sciences University of Florence Florence Italy; ^2^ Department of Statistics, Computer Science, Applications University of Florence Florence Italy; ^3^ Gastroenterology Unit, Department of Experimental and Clinical Biochemical Sciences University of Florence Florence Italy; ^4^ Obstetrics and Gynaecology Unit, Department of Experimental and Clinical Biomedical Sciences University of Florence Florence Italy

**Keywords:** diagnostic thresholds, intrahepatic cholestasis of pregnancy, reference ranges, total serum bile acids

## Abstract

**Objective:**

To establish pregnancy‐specific reference ranges for fasting and postprandial total serum bile acid (TSBA) concentrations.

**Design:**

Cross‐sectional study.

**Setting:**

Tertiary‐care university hospital.

**Population:**

Healthy pregnant women at term admitted to the Obstetrics Department over a period of 1 year. Exclusion criteria were an established diagnosis of intrahepatic cholestasis of pregnancy (ICP) or any coexisting condition of increased risk for ICP.

**Methods:**

Both fasting (after 8–14 h of fasting) and postprandial (2 h after meal) TSBA concentrations were measured in 612 women (with 528 fasting samples and 377 postprandial samples) by automated enzymatic spectrophotometric assay.

**Main outcome measures:**

Fasting and postprandial TSBA concentrations in 612 women.

**Results:**

Reference intervals of 4.4–14.1 μmol/L for fasting TSBA and 4.7–20.2 μmol/L for postprandial TSBA were established. The postprandial values were significantly higher than the fasting values, with a median increase of 1.0 μmol/L (*p* < 0.0001). A correlation between fasting TSBA concentrations and postprandial concentrations was found, as well as correlations with fetal sex, parity and assisted reproductive technologies. A seasonal pattern was noticed for both fasting and postprandial TSBA, with the highest values measured in the winter season (*p* < 0.01 and 0.02, respectively)

**Conclusions:**

Normal pregnancy is associated with mild hypercholanaemia, and therefore a higher threshold should be considered for the diagnosis of ICP. We suggest using the upper reference limits observed in our healthy pregnant population (14 μmol/L for fasting TSBA and 20 μmol/L for postprandial TSBA). As the fasting measurement is more specific for the diagnosis, and the postprandial measurement is essential for the assessment of severity, it is recommended to measure both values rather than use random sampling.

**Tweetable abstract:**

Normal pregnancy is associated with mild hypercholanaemia, a higher threshold should be considered for the diagnosis of ICP.

## Tweetable abstract

Normal pregnancy is associated with mild hypercholanaemia, a higher threshold should be considered for the diagnosis of ICP.

**Linked article**: This article is commented on by Ovadia et al., pp. 1897–1898. in this issue. To view this minicommentary visit https://doi.org/10.1111/1471‐0528.17171

## 1 INTRODUCTION

Intrahepatic cholestasis of pregnancy (ICP) is the most common pregnancy‐associated liver disease. It classically presents in the third trimester, the cardinal clinical symptom is pruritus and the specific laboratory examination is an elevated level of total serum bile acids (TSBA).^1^, ^2^

The prevalence of the condition is 0.2%–2.0%, but varies greatly among ethnic groups and geographic regions. The pathogenesis of ICP is multifactorial: environmental factors, genetic predisposition and underlying liver disease, combined with the physiological increase of reproductive hormone synthesis in pregnancy, contribute to the development and severity of the disease.^1^, ^3^, ^4^, ^5^, ^6^ ICP is a relatively nonthreatening condition to the mother, but is associated with fetal complications such as spontaneous preterm delivery, meconium passage and, in severe cases, intrauterine death.^7^ The increased risk of stillbirth is possibly linked to the toxicity of bile acids on fetal cardiomyocytes or to the vasoconstriction of chorionic vessels.^8^, ^9^, ^10^

Internationally, there is lack of consensus regarding the diagnostic criteria for ICP. Most guidelines agree on the requirement of pruritus accompanied by otherwise unexplained abnormal liver function, both of which resolve rapidly after delivery. The TSBA level is the most often used biomarker for the diagnosis.^11^, ^12^, ^13^

The Royal College of Obstetricians and Gynaecologists (RCOG) guideline recommends that the upper limit of pregnancy‐specific ranges of TSBA should be used for diagnosis.^14^ Nevertheless, the reference ranges used in clinical laboratories are most often fasting values measured in nonpregnant subjects, a result of extremely limited data for pregnancy‐specific reference ranges.^4^, ^13^, ^15^, ^16^ Consequently, there is a wide range of diagnostic criteria for ICP. Most guidelines recommend the use of TSBA values of 10–15 μmol/L as a diagnostic threshold, but this may be reduced to 6–10 μmol/L in fasting women.^1^, ^11^, ^12^ In clinical practice, the most widely used threshold is non‐fasting 10 μmol/L, despite not being supported by strong evidence.^11^, ^16^

Recently, only extremely high TSBA concentrations (≥100 μmol/L) were shown to markedly increase the risk of stillbirth.^17^ Nevertheless, as no pharmacological treatment has been shown to reduce the subsequent risk of stillbirth,^18^, ^19^, ^20^, ^21^ early delivery is still often recommended.^2^, ^7^, ^17^ Policies of active management result, however, in increased intervention, caesarean section rate and iatrogenic prematurity that must be balanced against possible reductions in perinatal mortality. The diagnosis of ICP has serious implications for maternal and especially fetal and neonatal health,^22^ and therefore a correct diagnosis is essential.

The aim of this study was to investigate values of fasting and postprandial TSBA in healthy pregnant women and to establish the reference standard in pregnancy.

## 2 METHODS

This is a cross‐sectional study of TSBA concentrations in pregnant women attending Florence Careggi University Hospital, a tertiary referral maternity hospital. Healthy pregnant women at term admitted to the Obstetrics Department between 2020 and 2021 were offered participation. The reference population was defined as ‘healthy’ after excluding women with any pathology for which there is an association with the measurement being considered.

The inclusion criteria were: singleton pregnancy; gestational age of ≥37 weeks; and body mass index (BMI) of 17–40 kg/m^2^. We chose to include only full‐term pregnant women to restrict the gestational window of TSBA evaluation, making the sample more homogeneous.

Exclusion criteria were the presence of an established diagnosis of ICP or abnormal liver function tests at any time throughout the pregnancy. We also excluded any coexisting condition with an increased risk for ICP, such as: multiple pregnancy; personal history of ICP; personal history of liver disease (such as a history of hepatitis B or C); cholecystectomy; history of gastric bypass surgery; and an inability to provide informed consent. None of the women had pruritus or abnormal liver function at the time of the TSBA assessments.

Both fasting (measured at 8:00 am, after 8–14 h of fasting) and postprandial (measured at 2:00 pm, 2 h after a meal) TSBA were measured. The limited time frame in which the blood samples could be sent to the laboratory, as well as the dynamic nature of the obstetrics department, was the main limit to patient inclusion. Not all potential candidates eligible for the study could participate or give informed consent. In particular, pregnant women who were sent to the delivery room before a blood sample was taken could not participate.

For each patient both venous blood samples were collected, whenever possible, in accordance with the needs of the laboratory (as specified above); otherwise, only one of the two blood samples was taken.

TSBA concentrations correspond to the sum of more than 20 individual bile acids,^23^ and were estimated by automated enzymatic spectrophotometric assay, based on microbial 3α‐hydroxysteroid dehydrogenase. Blood samples were analysed using the Total Bile Acids Assay Kit (Sentinel Diagnostics, Milan, Italy) at the Careggi Hospital clinical laboratory.

TSBA values were used for reference interval calculation, according to the recommendations of the International Federation of Clinical Chemistry and Clinical and Laboratory Standards Institute, C28‐A3. We aimed to include at least 120 samples for both fasting and postprandial TSBA, which is the minimum number of samples suggested for determining reference levels and confidence intervals.^24^ In order to evaluate the seasonal pattern, and to have the same number of patients enrolled in each season, a patient's enrolment continued until reaching 12 months of recruitment.

An abnormal level was defined as a value exceeding the upper reference limit (97.5th percentile),^25^, ^26^ as there is no known clinical significance for low concentrations of TSBA. In our laboratory, the normal range of TSBA in the general population lies between 0 and 6 μmol/L.

The laboratory results were collected in a database along with maternal and pregnancy characteristics. This information was obtained upon admission, as part of the information routinely collected for hospitalisation. Patients were then followed up until delivery, and data regarding the delivery and neonatal outcome were collected.

### 2.1 Statistical analyses

Continuous variables were represented using mean, standard deviation, median, minimum and maximum values, whereas categorical variables were represented using absolute and relative frequencies. In order to calculate the reference interval for fasting and postprandial TSBA the nonparametric percentile method was used. For each parameter the 2.5th and 95th percentiles and 95% confidence intervals were reported.

Pearson's correlation coefficient was used to assess the association between fasting or postprandial TSBA and maternal and neonatal characteristics. To evaluate the difference in fasting or postprandial TSBA between groups a Student's *t*‐test, Satterthwaite *t*‐test or a Mann–Whitney *U*‐test were used, according to the Shapiro–Wilk test and *F*‐test for normality and homoscedasticity, respectively.

The Kruskal–Wallis test was used to assess the difference in fasting or postprandial TSBA between seasons, according to the Shapiro–Wilk test for normality.

The statistical analyses were conducted using the statistical software package sas 9.3 and the significance level was set at 5%.

## 3 RESULTS

A total of 612 women participated in the study, including 525 white women, 50 South‐Asian women, 27 Hispanic women and 10 Sub‐Saharan African women. We collected 528 fasting samples and 377 postprandial samples. For 293 women both samples were taken, exceeding the minimum suggested 120 samples for determining reference intervals and confidence intervals according to the Clinical Laboratory and Standards Institute.^24^

The indication for admission of the patients was divided into nine main groups: elective Caesarean section (*n* = 149), premature rupture of the membranes (*n* = 44), latent phase of labour (*n* = 57), gestational hypertension (*n* = 53), gestational diabetes mellitus (*n* = 80), maternal indications (*n* = 58), fetal indications (*n* = 75), amniotic fluid anomalies (*n* = 59) and post‐term pregnancy (*n* = 37). TSBA concentration did not differ between the nine groups (*p* = 0.8 for fasting TSBA and *p* = 0.37 for postprandial TSBA). In the whole cohort there were no cases of chorioamnionitis, fever or abnormal cardiotocography.

**FIGURE 1:**
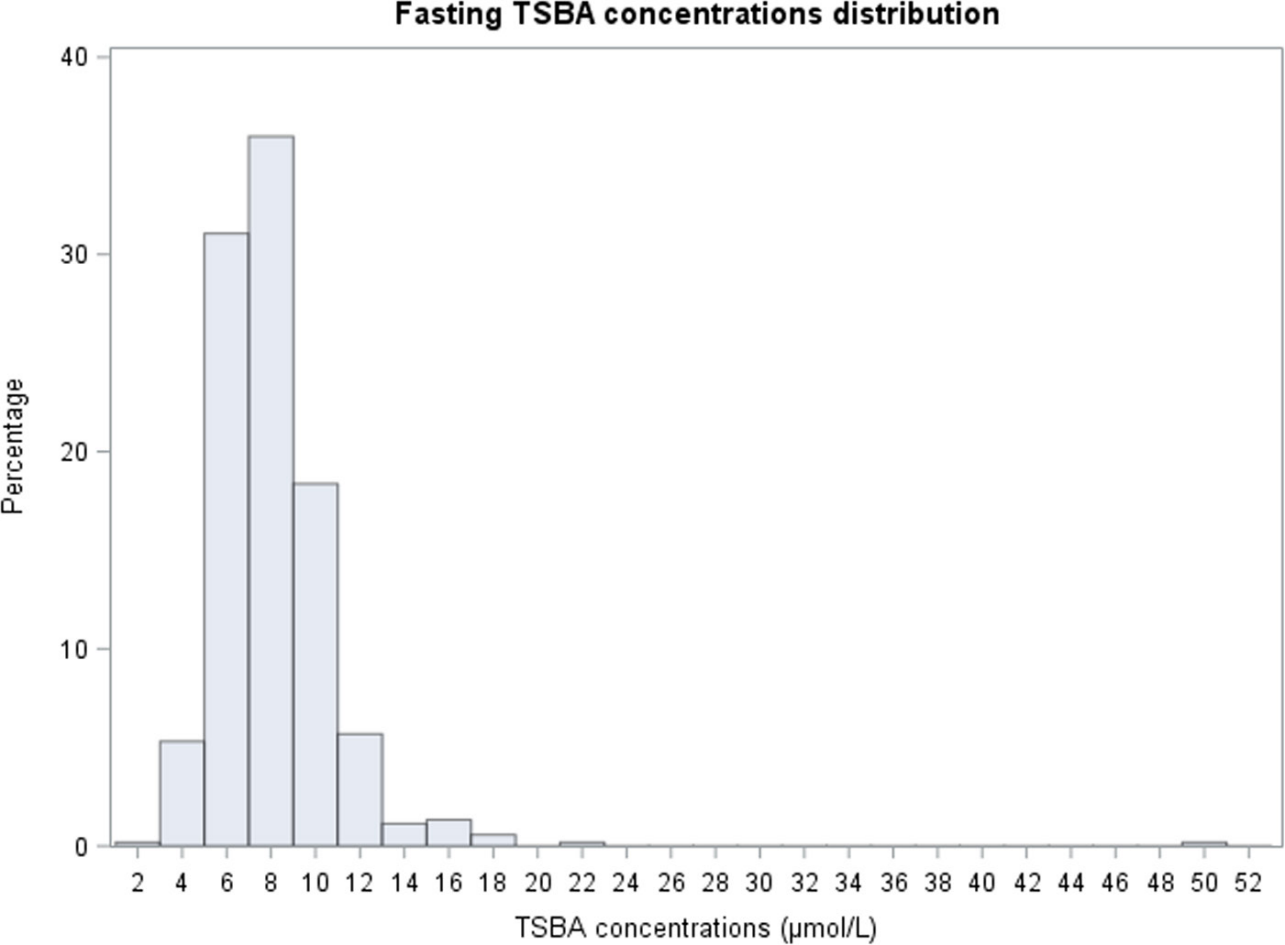
Fasting TSBA concentration distribution

**FIGURE 2:**
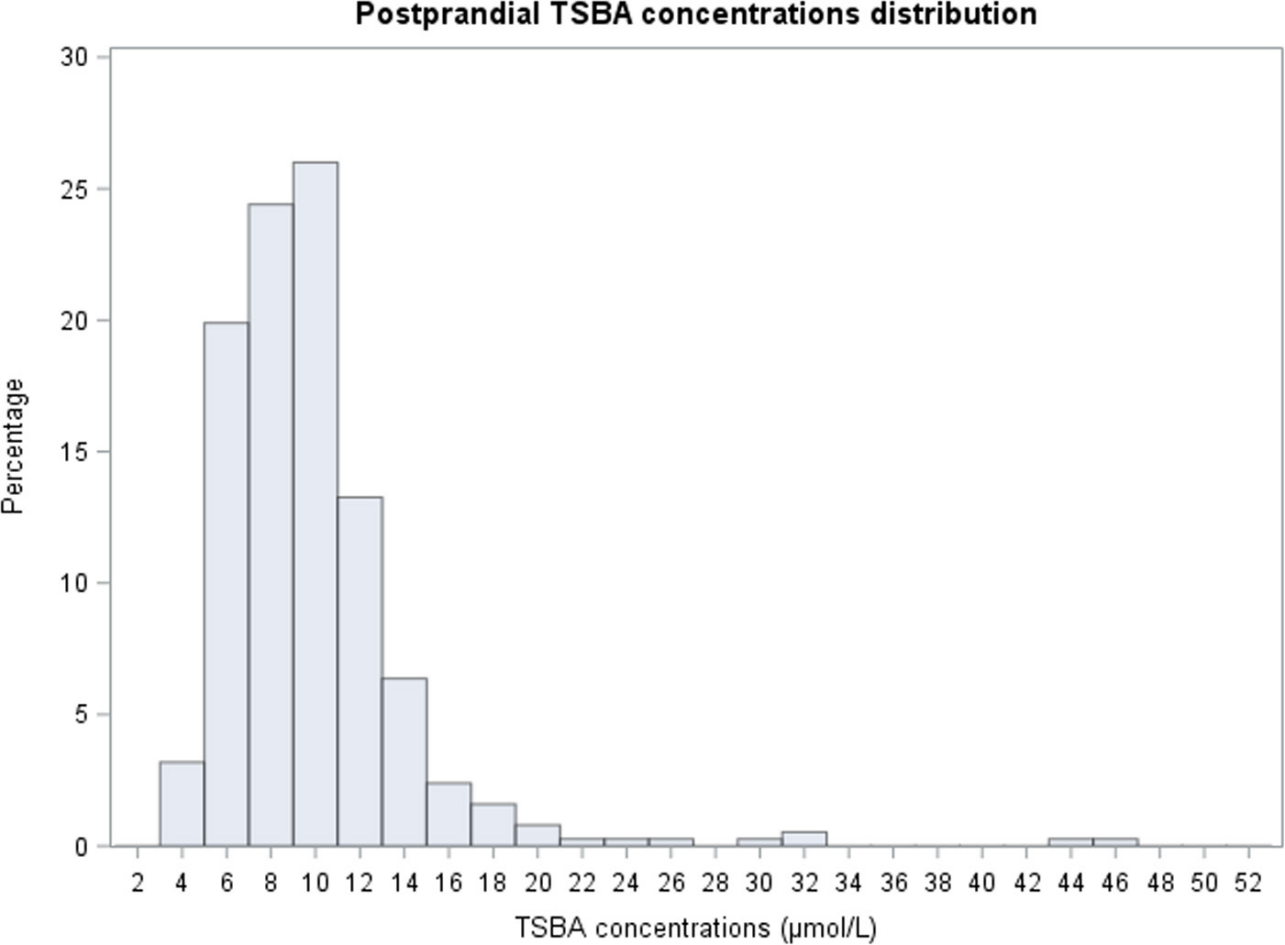
Postprandial TSBA concentration distribution

In our analysis, we found that the TSBA concentrations for both groups of samples were not normally distributed but instead had a positively skewed distribution, in accordance with the findings of other studies (Figures 1 and 2).^27^, ^28^ We found a median of 7.6 μmol/L for the fasting TSBA, with an upper reference limit (97.5th percentile) of 14.1 μmol/L (95% CI 12.7–15.5 μmol/L). We found a median TSBA concentration of 9.1 μmol/L in the postprandial samples, with an upper reference limit of 20.2 μmol/L (95% CI 17.3–32.3 μmol/L). We established reference intervals of 4.4–14.1 μmol/L for fasting TSBA and 4.7–20.2 μmol/L for postprandial TSBA (Table 1).

**TABLE 1:** Maternal characteristics and reference interval calculations of TSBA (fasting and postprandial)

Age (years)^b^	34 (30, 38)	*N* = 612
BMI (kg/m^2^)^b^	22.71 (20.6, 25.6)	*n* = 610
Gestational weight gain (kg)^b^	12 (9, 15)	*n* = 602
Neonatal weight (g)^b^	3268.9 (±466.47)	*n* = 611
Fasting TSBA reference (μmol/L)	7.6 (6.4, 9.1)	*n* = 528
Lower limit (95% CI)^a^	4.4 (4.2–4.7)	
Upper limit (95% CI)^a^	14.1 (12.7–15.5)	
Postprandial TSBA reference (μmol/L)^b^	9.1 (7.1–11)	*n* = 377
Lower limit (95% CI)^a^	4.7 (4.5–5.3)	
Upper limit (95% CI)^a^	20.2 (17.3–32.3)	

^a^ Reference intervals were calculated using nonparametric method to account for non‐normally distributed results. The lower (2.5th percentile) and upper (97.5th percentile) reference limits are noted with 95% confidence intervals (95% CIs) indicated in brackets.

^b^ Data are expressed as means ± SDs or median (first, third interquartile range), based on their distribution.

When applying the currently used thresholds to our asymptomatic pregnant population, we found that TSBA concentrations exceeded 10 μmol/L, the most commonly used threshold, in 15.7% of the fasting measurements and in 38.5% of the postprandial measurements. The fasting threshold of ≥6 μmol/L was present in 83.3% of the pregnant population, and the postprandial threshold of ≥15 μmol/L was present in 6.9% of the pregnant population.

It is interesting to note that although not of clinical relevance, our lower reference limit is higher than that previously reported. These results suggest that pregnancy‐specific reference ranges are shifted to the right with respect to those of the nonpregnant population.

When both measurements were performed (293 patients), the postprandial values were significantly higher than the fasting measurement, with a median increase of 1.0 μmol/L (95% CI 0.7–1.3 μmol/L; *p* < 0.0001). On correlation analysis, fasting TSBA concentrations were moderately correlated with postprandial TSBA concentrations (Pearson's coefficient 0.44; *p* < 0.0001) (Tables 2 and 3).

**TABLE 2:** Correlation between TSBA concentrations and maternal and neonatal characteristics

	Characteristics	Pearson's CC	*p*
Fasting TSBA	Maternal BMI	−0.05	0.2200
	Weight gain	−0.01	0.7737
	Age	0.07	0.1154
	Neonatal weight	0.08	0.0771
	Postprandial TBSA	**0.44**	**<0.0001**
Postprandial TSBA	Maternal BMI	−0.06	0.2784
	Weight gain	−0.01	−0.8802
	Age	0.08	0.1381
	Neonatal weight	0.07	0.1653

Bold values are statistically significant (*p*‐value < 0.05).

**TABLE 3:** Correlation between fasting TSBA concentrations and maternal and neonatal characteristics

Fasting TSBA	Number	Median	Q1	Q3	*p*
Nulliparous	314	7.65	6.6	9.4	**0.0417**
Multiparous	214	7.40	6.3	8.8	
No progestinic therapy	407	7.60	6.4	9	0.5316
Progestinic therapy	121	7.50	6.5	9.4	
No gestational diabetes	398	7.60	6.4	9.2	0.1534
Gestational diabetes	130	7.45	6.3	8.9	
No hypertensive disorders	452	7.55	6.4	9.1	0.7664
Hypertensive disorders	76	7.65	6.3	9.2	
No intrauterine growth restriction	461	7.60	6.4	9.2	0.0620
Intrauterine growth restriction	66	7.25	6.3	8.7	
No hypothyroidism	411	7.60	6.4	9.1	0.8180
Hypothyroidism	117	7.50	6.4	9.1	
No assisted reproductive technologies	466	7.50	6.3	9	**0.0164**
Assisted reproductive technologies	62	8.45	6.8	9.8	
Female fetus	260	7.2	6.2	8.8	**0.0002**
Male fetus	267	8.1	6.6	9.5	
Indication for admission					
Elective caesarean section	148	7.55	6.3	9.6	0.8072
Latent phase of labour	31	7.5	6.6	8.9	
Premature rupture of membranes	41	7.5	6.3	9.1	
Hypertensive disorders	48	7.6	6.65	9.4	
Gestational diabetes	66	7.4	6.1	8.4	
Maternal indication	48	7.65	6.4	9	
Fetal indication	65	7.6	6.1	9.1	
Post‐term pregnancy	28	8.25	6.55	9.5	
Amniotic fluid anomalies	53	7.7	6.6	9.4	

Bold values are statistically significant (*p*‐value < 0.05).

We observed higher fasting TSBA concentrations in pregnancies achieved with the use of assisted reproductive technology (ART), compared with spontaneous conception (median 8.45 and 7.5 μmol/L, respectively; *p* = 0.02). The reasons for the association between ART and higher TSBA concentrations are unclear, but they could be related to some metabolic disturbance linked to the infertility itself or the hormonal maintenance therapy used for ART.^29^ We also noticed higher fasting TSBA concentrations in nulliparous compared with multiparous women (*p* = 0.04), although the reason for this association is unclear.

Interestingly, we also found a correlation between fasting TSBA and fetal sex, with the male sex being associated with higher concentrations of TSBA (8.1 vs 7.2 μmol/L; *p* < 0.001). To our knowledge, this is the first time a correlation with fetal sex has been made and can be explained by differences in hormonal metabolism by the fetal liver. The magnitude of the observed differences, however, may not be clinically relevant.

Fasting TSBA concentrations were not correlated with maternal age, BMI, weight gain and neonatal birthweight, and no association was found with gestational diabetes mellitus, hyperthyroidism, hypertensive disorders, intrauterine growth restriction (IUGR) or progesterone therapy (Tables 2 and 3).

We found that postprandial TSBA values were significantly lower in pregnancies complicated by IUGR (median 7.8 vs 9.15 μmol/L in non‐IUGR fetuses; *p* = 0.03), although this difference is difficult to explain and may not be clinically relevant. Postprandial values were not associated with any of the other variables examined (Tables 2 and 3).

**FIGURE 3:**
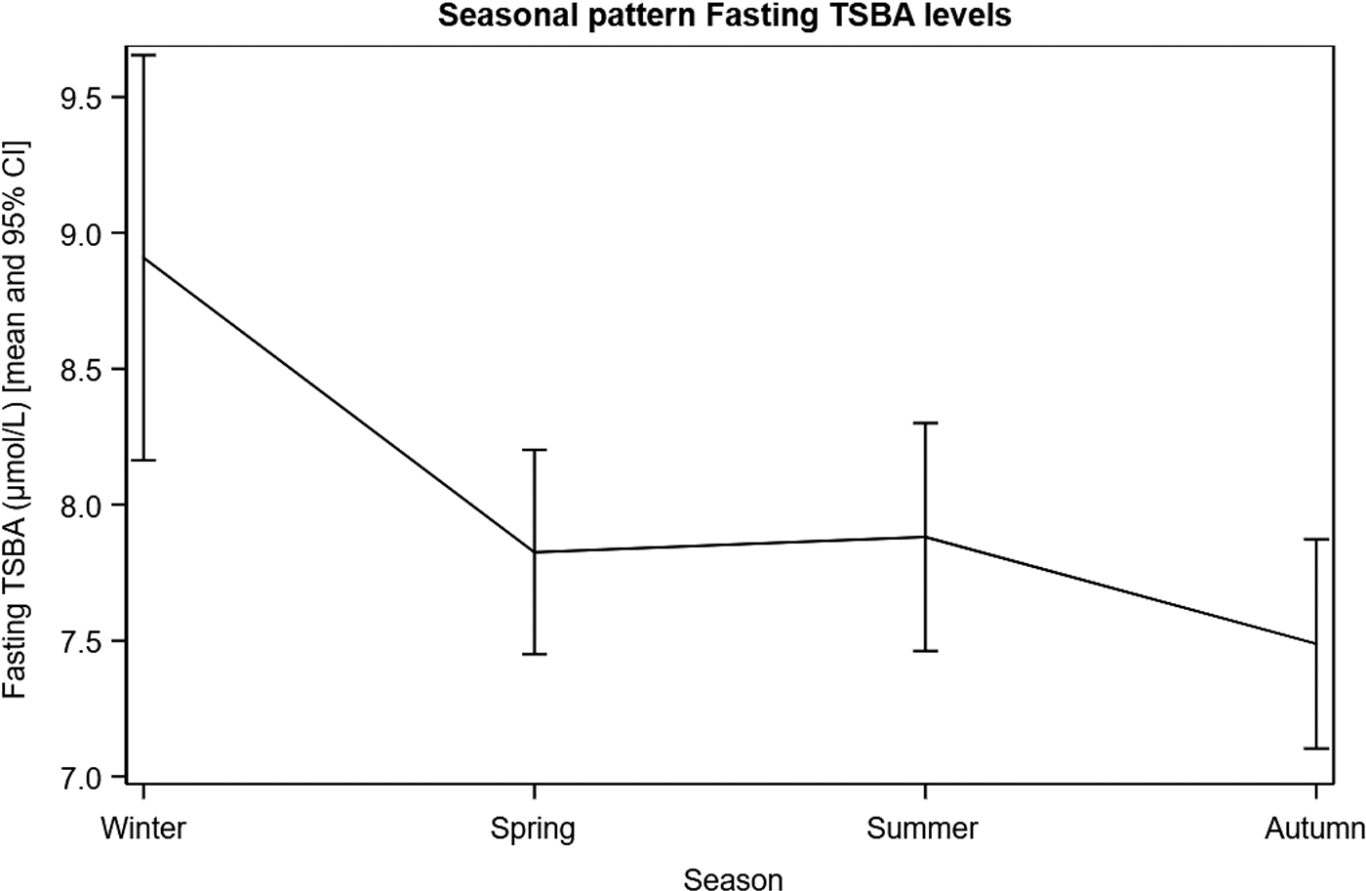
Seasonal pattern of fasting TSBA levels

**FIGURE 4:**
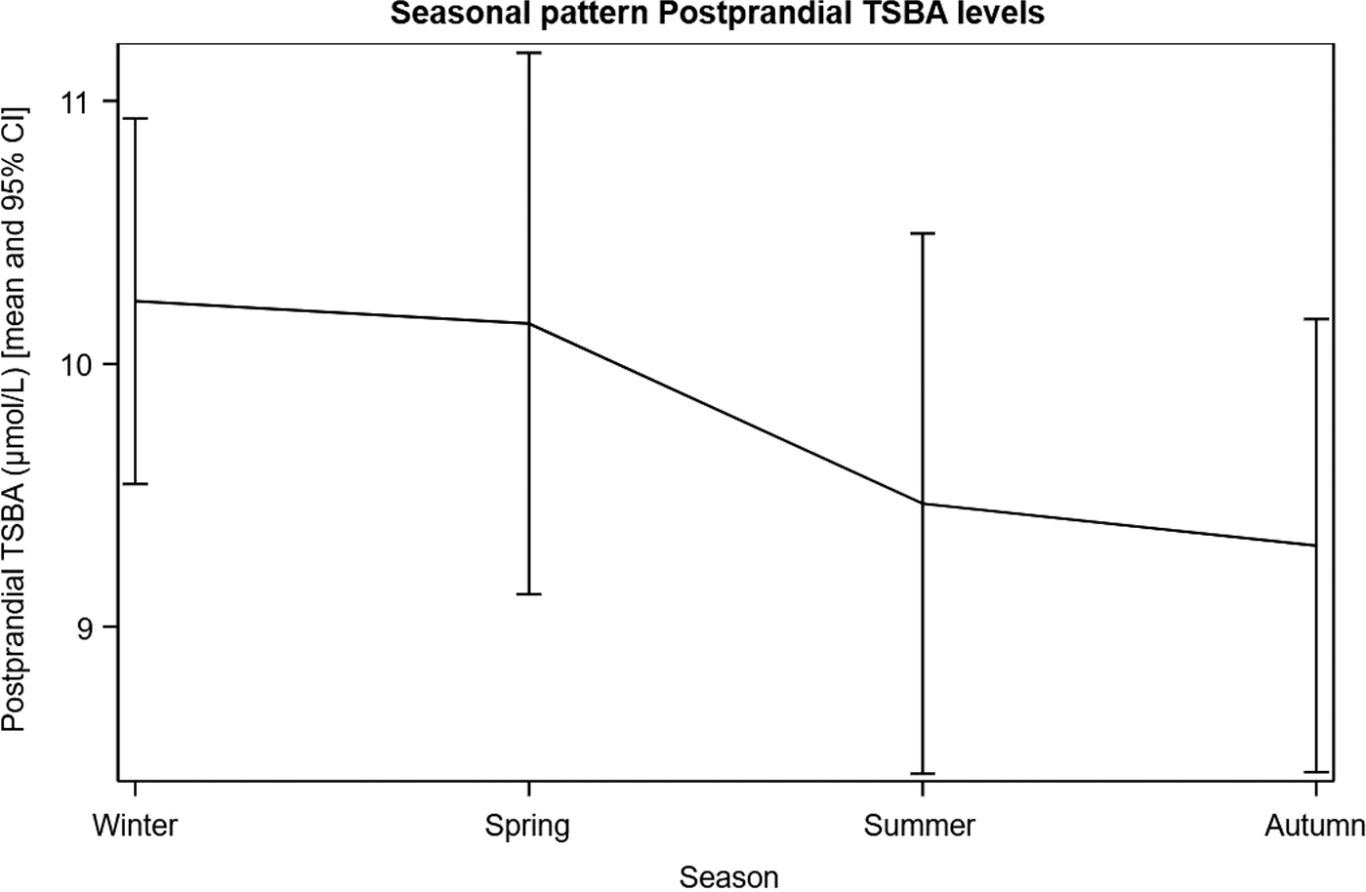
Seasonal pattern of postprandial TSBA levels

We noticed a seasonal pattern for both fasting and postprandial TSBA concentrations, with the highest values measured in the winter season, declining during spring and summer, and with minimum values measured in the autumn (*p* < 0.01 and 0.02, respectively) (Figures 3 and 4).

Intrahepatic cholestasis of pregnancy (ICP) is known to be more common in South America and in Northern Europe.^5^ Nevertheless, we did not find a strong correlation with ethnicity, probably because the majority of patients in our study were white (Table 4).

**TABLE 4:** Correlation between fasting TSBA concentrations and maternal and neonatal characteristics

Postprandial TSBA	Number	Median	Q1	Q3	*p*
Nulliparous	248	9.25	7.2	11.25	0.4188
Multiparous	129	8.80	7.1	10.8	
No progestinic therapy	297	9.10	7.2	11.2	0.6281
Progestinic therapy	80	9.00	6.95	10.9	
No gestational diabetes	277	9.20	7.4	11.2	0.1734
Gestational diabetes	100	8.80	6.6	10.9	
No hypertensive disorders	319	9.00	7.1	11	0.3314
Hypertensive disorders	58	9.35	7.5	12	
No intrauterine growth restriction	334	9.15	7.3	11	**0.0321**
Intrauterine growth restriction	42	7.80	6.1	11	
No hypothyroidism	287	9.00	7.1	11.2	0.7440
Hypothyroidism	90	9.15	7.2	10.9	
No assisted reproductive technologies	337	9.00	7.1	11	0.4208
Assisted reproductive technologies	40	9.65	7.45	11.95	
Female fetus	174	9.00	6.9	11	0.4810
Male fetus	202	9.10	7.3	11.3	
Indication for admission					
Elective caesarean section	18	8.4	7	10.5	0.3571
Latent phase of labour	29	8.6	6.5	10.2	
Premature rupture of membranes	42	9.85	7.9	11.5	
Hypertensive disorders	40	9.2	7.95	12.35	
Gestational diabetes	71	8.6	6.5	10.5	
Maternal indication	51	9.2	7.7	10.8	
Fetal indication	55	9.2	6.5	11.3	
Post‐term pregnancy	27	9.6	6.7	11.7	
Amniotic fluid anomalies	44	9.4	8.15	11.8	

Bold values are statistically significant (*p*‐value < 0.05).

## 4 DISCUSSION

### 4.1 Main findings

In our study we aimed to establish pregnancy‐specific reference ranges for fasting and postprandial TSBA concentrations. Despite using restrictive exclusion criteria, we demonstrated that the normal reference range and the upper reference limit in pregnancy differ from those established for the general nonpregnant population. Compared with the reference range for normal adults (0.28–6.5 μmol/L),^23^, ^30^, ^31^ we found significantly higher fasting TSBA (4.4–14.1 μmol/L) and postprandial TSBA (4.7–20.2 μmol/L) reference intervals in the pregnant population.

Our study confirms that TSBA concentrations increase in response to food intake and that differences between fasting and postprandial measurements are clinically and statistically significant.^13^, ^16^

### 4.2 Strengths and limitations

The main strength is the large cohort of pregnancies analysed (612 patients), which to our knowledge is the largest sample of healthy pregnant women that has been examined for TSBA so far.

We believe the strengths also include the prospective design, the accurate selection of eligible patients, the generalisability and the clinical relevance of the results. As no correlation has been reported between TSBA concentrations and gestational age within the third trimester,^16^, ^27^ our results could be applicable to the third trimester of pregnancy. The duration of the study over 1 year allowed us to evaluate the seasonality of TSBA concentrations. Finally, all blood samples were analysed in the same laboratory and were taken as needed for study purposes.

The low ethnic diversity in the study reflects that most patients in our hospital are white and the difficulty in collecting informed consent from non‐Italian speakers. This limited our ability to evaluate variations in normal TSBA concentrations between different ethnic groups. In addition, the external validity of our findings may be limited by the inclusion of patients from a single institution. Although widely used in clinical laboratories, the most important limitation of the enzymatic method used is the low sensitivity, as the lowest concentration of TSBA measurable is around 1.5 μmol/L.^16^, ^22^ Nevertheless, 100% of the measured values were above the evaluation limit. Another limitation is that hospital meals were not standardised for macronutrients and potentially differed across the cohort tested.

### 4.3 Interpretation

Evidence that normal pregnancy may be associated with a mild sub‐cholestatic state has been described in previous studies. Although based on a limited number of patients, an increase has been demonstrated in the mean values of single bile acid and total bile acid concentrations in uncomplicated pregnancies with no other evidence of ICP.^32^, ^33^, ^34^, ^35^

Our results are consistent with early observations that pregnancy is associated with mild hypercholanaemia,^32^, ^33^, ^34^, ^35^ and this can be explained by the cholestatic effect of reproductive hormones. Normal pregnancy is a hyperestrogenic state and is therefore associated with a physiological elevation of TSBA. Thus, obstetricians need to be aware that healthy pregnant women have increased concentrations of TSBA when assessing women who may have ICP.

Early studies have described that ICP has a seasonal pattern, with increased incidence in some countries during the winter months, suggesting a possible association with an environmental trigger. This pattern, however, has not yet been demonstrated in clinical studies. Two possible explanations are low levels of natural selenium and vitamin D during the winter, as both deficiencies have been reported in women with ICP.^1^, ^5^, ^36^, ^37^

It has recently been suggested that higher thresholds should be used for the diagnosis of ICP, given the low risk of stillbirth demonstrated recently for TSBA concentrations of <100 μmol/L.^16^, ^27^

The assessment of diagnostic criteria for ICP is complex, as the classic methodologies used to establish diagnostic thresholds for other pathologies are difficult to apply here. Currently, in clinical practice, ICP is diagnosed by elevated TSBA concentrations that are measured after the onset of pruritus, a non‐specific symptom that is not necessarily associated with ICP. Thus, the diagnosis is based on TSBA alterations (as pruritus without TSBA elevation will not be diagnosed as ICP) by using nonpregnant reference ranges. In nonpregnant patients, TSBA concentrations are used as a biomarker for hepatic injury, whereas in pregnant patients the outcome of interest is not liver dysfunction but rather the absolute elevation in circulating TSBA concentrations.^4^ Besides pruritus, TSBA elevation is not associated with adverse outcome until it reaches severely increased concentrations (>100 μmol/L for stillbirth and >40 μmol/L for other adverse outcomes), and thereby the diagnostic threshold cannot be tested by maternal or neonatal outcomes.

For these reasons, an important limitation when studying the normal distribution of TSBA concentrations in pregnancy is that the accuracy (sensibility and specificity) of the upper limit value of normality in the ICP population cannot be calculated, as ICP was originally diagnosed by the same criteria that need to be tested.

Our results demonstrate that the nonpregnant reference range cannot be used in the pregnant population. In accordance with the RCOG clinical guidelines for ICP,^14^ we suggest using the upper limit value of our normal pregnancy‐specific reference range in clinical practice. Specifically, 14 μmol/L for fasting TSBA values and 20 μmol/L for postprandial TSBA values. A similar threshold (19 μmol/L) was suggested by a recent study that re‐evaluated the diagnostic thresholds for ICP. Although determined using different methodologies, our postprandial threshold was similar to the random threshold described by Mitchell et al.^27^ In accordance, we also suggest that patients with otherwise unexplained pruritus with TSBA concentrations below the proposed thresholds should repeat the TSBA measurements, as pruritus often precedes an elevation in TSBA concentrations.^2^, ^38^

There is continuing debate regarding which value should be used to diagnose ICP in clinical practice. Postprandial TSBA assessment may be a more sensitive test, whereas elevated fasting TSBA concentrations are a more specific indicator of severe liver disease.^13^, ^14^ Some authors suggest measuring TSBA concentrations in the fasting state because of the large overlap between normal non‐fasting values and the commonly used thresholds for diagnosis.^16^ On the other hand, considering that the adverse perinatal outcomes of ICP are associated with peak TSBA concentration, a non‐fasting measurement has greater clinical relevance.^27^

We suggest using both values of TSBA. We recommend distinguishing the fasting and postprandial status and avoiding random sampling because of the different reference ranges and upper limit values that should be applied. Fasting TSBA concentrations may be the most specific for the diagnosis of ICP and therefore better for confirming ICP. Fasting measurements are also more predictable, have less variability and correlate better with certain risk factors. The postprandial measurement, on the other hand, is essential for risk stratification. We suggest using postprandial TSBA concentrations to assess the severity of the disease and to follow‐up patients with ICP for subsequent management of the pregnancy and eventual active management.

## 5 CONCLUSION

In our study we demonstrated that, compared with nonpregnant adults, pregnancy is associated with mild hypercholanaemia. By defining pregnancy‐specific reference ranges we can avoid the unnecessary diagnosis of ICP, which is strongly correlated with maternal anxiety and active management.

We recommend that a higher threshold should be used for the diagnosis of ICP. We suggest using the upper limit in the pregnant population: fasting TSBA values of ≥14 μmol/L and postprandial TSBA values of ≥20 μmol/L. We also suggest that both values should be measured, as each provides different information: the fasting measurement is more specific for the diagnosis and the postprandial measurement is essential for risk stratification and severity assessment. As TSBA values usually increase after food intake, the measurement should not be random, as two different thresholds should be used for the fasting and postprandial measurements.

## CONFLICT OF INTERESTs

None declared. Completed disclosure of interests form available to view online as supporting information.

## AUTHOR CONTRIBUTIONS

MH and CL performed data curation, investigation and wrote the article, together with VS. VS also provided a critical review and text editing. LT performed the data analysis. MDT was the project administrator, conceived the idea and designed the study. MDT, FP and AG provided validation and visualisation of the final article.

## ETHICAL APPROVAL

This study was performed according to the principles of the Declaration of Helsinki (2013) and was approved by the ethics committee of Careggi University Hospital (ref. no. 18008_bio; 18 December 2020).

## Supporting information


ICMJE

ICMJE

ICMJE

ICMJE

ICMJE

ICMJE

ICMJE

## Data Availability

The data that support the findings of this study are available from the corresponding author, upon reasonable request.
